# A patient-aware benchmarking of CNN and transformer architectures for breast cancer histopathology classification

**DOI:** 10.3389/fdgth.2026.1752938

**Published:** 2026-05-08

**Authors:** Veeram Priyanka, Modigari Narendra, Tharasi Dilleswar Rao

**Affiliations:** 1 School of Advanced Sciences, Vellore Institute of Technology, Chennai, India; 2 School of Computer Science and Engineering, Vellore Institute of Technology, Chennai, India

**Keywords:** BreakHis dataset, breast cancer histopathology, computational pathology, ConvNeXt, deep learning benchmarking, patient-aware cross-validation, statistical significance analysis, swin transformer

## Abstract

**Introduction:**

Breast cancer diagnosis using histopathological imaging remains a critical yet challenging task, requiring automated systems that generalize reliably across patients and varying imaging conditions. While deep learning models have shown strong performance, many prior studies employ image-wise data splits that introduce patient-level data leakage, resulting in overly optimistic and potentially misleading evaluations. This study aims to address this limitation by establishing a rigorous, leakage-free benchmarking framework for binary breast cancer histopathology classification.

**Methods:**

A comprehensive evaluation of nine deep learning architectures was conducted on the BreaKHis dataset, comprising 7,909 images from 82 patients. The models include six convolutional neural networks (ResNet50, MobileNetV2, VGG16, DenseNet121, Xception, and EfficientNetB0), one modern convolutional architecture (ConvNeXt), and two transformer-based models (Swin-Small and Swin-Base). A strict 5-fold patient-aware cross-validation protocol was implemented to ensure that images from the same patient were not shared between training and validation sets. All models were trained under identical experimental conditions. Performance was assessed using accuracy, precision, recall, and F1-score, reported as mean ± standard deviation. Statistical significance was evaluated using paired *t*-tests and Wilcoxon signed-rank tests with Bonferroni correction.

**Results:**

All evaluated architectures demonstrated comparable performance, achieving mean accuracies in the range of 0.91-0.93. ResNet50 achieved the highest mean accuracy (0.9267 ± 0.0435) and F1-score (0.9472), although differences among models were marginal. Statistical analysis confirmed that no pairwise differences were statistically significant (*p* > 0.05 after correction). Magnification-wise analysis indicated that intermediate resolutions (40× and 200×) provided more discriminative features, whereas higher magnification (400×) resulted in reduced performance due to limited contextual information.

**Discussion:**

The findings highlight that, under a rigorously controlled and leakage-free evaluation protocol, architectural differences among modern deep learning models do not lead to statistically significant performance variations. Instead, evaluation design plays a more critical role in determining reliable outcomes. The proposed patient-aware benchmarking framework enhances reproducibility and provides a robust foundation for future research, supporting the development of clinically translatable AI systems for breast cancer diagnosis.

## 1 Introduction

Breast cancer remains one of the most prevalent and life-threatening diseases worldwide, necessitating accurate and reliable diagnostic methods for early detection and treatment planning. Histopathological examination of tissue biopsies is considered the gold standard for diagnosis; however, it is time-consuming, subjective, and dependent on expert interpretation. These challenges have motivated the development of automated computational approaches using deep learning to assist pathologists in improving diagnostic efficiency and consistency. Recent advances in deep learning, particularly convolutional neural networks (CNNs), have demonstrated strong performance in medical image analysis tasks, including breast cancer classification [1, 2]. Architectures such as ResNet50 [3], DenseNet121 [4], and EfficientNet [5] have shown the ability to capture hierarchical texture and morphological features from histopathological images.

However, CNNs are inherently constrained by localized receptive fields, which may limit their ability to capture long-range spatial dependencies and global tissue architecture [6, 7]. To address this limitation, vision transformers, such as the Swin Transformer [8], have been introduced. By leveraging self-attention mechanisms, transformers can more effectively model these broad spatial relationships [9]. Despite these developments, it remains unclear whether transformer-based models offer statistically superior performance consistently outperform in histopathology tasks under realistic evaluation settings.

A critical limitation in many existing studies is the reliance on image-wise data splitting, where patches extracted from the same patient may appear in both training and validation sets. This introduces data leakage and can lead to overly optimistic performance estimates, particularly in patch-based datasets such as BreaKHis. Consequently, reported accuracies may not reflect true generalization to unseen patients. Furthermore, prior work often evaluates models under inconsistent experimental settings, including varying hyperparameters, training protocols, and evaluation strategies, making fair comparison difficult. In addition, many studies report performance improvements without statistical validation, limiting confidence in whether observed differences are meaningful.

Another limitation lies in the lack of comprehensive and controlled benchmarking across diverse model families. Existing literature frequently evaluates either CNNs or transformer-based models in isolation, or compares a limited subset of architectures without systematically controlling for training conditions. As a result, the relative strengths and limitations of different architectural paradigms in histopathology classification remain insufficiently understood.

To address these gaps, this study presents a rigorous and controlled benchmarking of nine deep learning architectures, including six CNN-based models (ResNet50, MobileNetV2, VGG16, DenseNet121, Xception, and EfficientNetB0), a modern convolutional architecture (ConvNeXt), and two transformer-based models (Swin-Small and Swin-Base). All models are evaluated on the publicly available BreaKHis dataset using a strict 5-fold patient-aware cross-validation protocol, ensuring that images from the same patient do not appear across training and validation sets. A unified training strategy is applied across all models to enable fair comparison, and performance is reported using multiple evaluation metrics along with statistical significance testing.

## 2 Literature review

Automated analysis of breast cancer histopathology has undergone a significant transition from handcrafted feature-based systems to data-driven deep learning frameworks. Early approaches relied on explicit feature engineering, where morphological, textural, and color-based descriptors were extracted and classified using traditional machine learning algorithms [10, 11]. Although these methods provided interpretability, their performance was limited by their inability to capture hierarchical tissue organization and their sensitivity to variations in staining, magnification, and imaging conditions.

The adoption of convolutional neural networks (CNNs) marked a major advancement by enabling end-to-end feature learning. CNN-based models have been widely applied to histopathological image classification, including on the BreaKHis dataset [12], demonstrating strong performance through hierarchical representation learning [2, 7]. Subsequent efforts introduced multi-scale architectures to account for variations in magnification [7], as well as ensemble methods that combine complementary model predictions [13]. Despite these improvements, most CNN-based studies remain focused on optimizing predictive performance within specific experimental setups, often without systematically evaluating generalization across patients or ensuring comparability across architectures.

Recent developments have explored transformer-based architectures as a powerful alternative to traditional convolutional models. Foundational models like the Vision Transformer (ViT) [14] and its hierarchical variants, such as the Swin Transformer [8], have demonstrated an exceptional ability to model long-range dependencies and contextual relationships. While these models initially showed promising results in broad medical image classification and segmentation tasks [9, 15], subsequent research has increasingly shifted toward domain-specific architectures tailored for histopathology. To capture the unique morphological complexity of tissue architectures, researchers have explored specialized pre-training and hybrid designs. For instance, Wang et al. [16] introduced TransPath, a hybrid architecture combining a CNN with a modified transformer. By utilizing self-supervised learning on a massive corpus of unlabeled histopathological images, TransPath demonstrated that domain-specific feature embeddings are highly transferable. Addressing the vast spatial scale of digital pathology, Chen et al. [17] proposed the Hierarchical Image Pyramid Transformer (HIPT), which leverages two levels of self-supervised learning to model the inherent hierarchical structure of gigapixel whole-slide images. More recently, the comparative efficacy of these paradigms has been investigated specifically for breast cancer diagnostics. Anand and Khajuria [18] conducted a comparative study between a modern convolutional model (ConvNeXt V2) and a progressive vision transformer (Swin Transformer V2) for binary breast mass classification, finding that the attention-driven global feature modeling of Swin V2 effectively captured subtle morphological variations, outperforming ConvNeXt V2 with a peak accuracy of 0.985. Together, these studies underscore the significant potential of transformer-based architectures in digital pathology. However, existing literature frequently reports improvements under variable experimental conditions, impeding the ability to differentiate whether performance gains arise from architectural design or differences in training protocols and data partitioning strategies. This reinforces the critical necessity for rigorous, controlled benchmarking across a broader spectrum of models on standardized patch-based datasets, such as BreaKHis, to fully understand their comparative strengths in clinical decision-support systems. For whole-slide image (WSI) analysis, multiple instance learning (MIL) frameworks, such as the transformer-based TransMIL [19], learn to aggregate patch-level information into slide-level predictions. While effective for large-scale WSIs, their considerable computational complexity and reliance on massive annotated datasets limit their practical applicability to smaller, patch-based benchmarks.

Beyond instance aggregation, recent research has emphasized the importance of explicitly modeling spatial and structural relationships within tissue microenvironments. For example, SpatialQPFs [20] capture cell-to-cell interactions and spatial distributions, providing biologically interpretable representations. Similarly, entropy-based methods [21] quantify structural complexity in histopathological images, offering a complementary perspective to conventional feature learning. More recent architectures, such as SlideMamba [22], integrate graph-based representations with sequence modeling techniques to enhance contextual understanding across spatial scales. These approaches highlight a shift toward incorporating higher-order structural information, which is not explicitly modeled in standard CNN or patch-based transformer frameworks.

Another emerging direction is the development of foundation models for computational pathology. These models leverage large-scale pretraining to learn transferable representations that can generalize across tasks and datasets. Recent work [23] demonstrates that such models can achieve strong performance across diverse histopathological benchmarks. However, their reliance on large-scale data and computational resources presents practical limitations, particularly for controlled experimental settings and smaller datasets such as BreaKHis.

Despite the diversity of approaches, several key limitations persist across the literature. First, many studies focus on specific architectural innovations without providing controlled comparisons across different model families. Second, inconsistencies in preprocessing, training protocols, and evaluation strategies hinder reproducibility and fair benchmarking. Third, statistical validation of performance differences is rarely conducted, limiting the ability to draw reliable conclusions regarding model superiority. Finally, while advanced paradigms such as MIL, spatial modeling, and foundation models offer improved representational capabilities, their complexity often restricts their use in standardized benchmarking scenarios.

In summary, while prior work has demonstrated the effectiveness of deep learning for histopathological classification, there remains a clear need for a systematic and controlled evaluation framework that enables fair comparison across diverse architectures. Such a framework is essential for understanding whether observed performance differences are attributable to model design or experimental variability.

## 3 Dataset description and preprocessing

This study utilizes the publicly available Breast Cancer Histopathological Image Classification (BreaKHis) dataset [12], a widely used benchmark for automated breast cancer diagnosis. The dataset comprises 7,909 hematoxylin and eosin (H&E)-stained histopathological images collected from 82 patients through surgical biopsies. The images are categorized into two classes: benign (2,480 images) and malignant (5,429 images). A key characteristic of BreaKHis is its multi-magnification structure, where each tissue sample is captured at four magnification levels: 40×, 100×, 200×, and 400×. These magnifications provide complementary diagnostic information across spatial scales. Lower magnifications (40× and 100×) capture global tissue architecture and stromal organization, whereas higher magnifications (200× and 400×) emphasize cellular morphology, including nuclear structure and mitotic activity. This variability introduces significant intra-class diversity, making the dataset suitable for evaluating model robustness under varying visual conditions.

To ensure clinically realistic evaluation and eliminate data leakage, the dataset was organized at the patient level. Each image is associated with a unique patient identifier extracted from the dataset structure, enabling grouping of correlated samples. A total of 82 patient groups were used as grouping variables in a 5-fold cross-validation scheme implemented via GroupKFold. In this setup, all images belonging to a given patient are assigned exclusively to either the training or validation set within each fold. This prevents patient-level leakage, where correlated samples from the same patient could otherwise appear in both sets and artificially inflate performance.

All images were resized to a fixed resolution of 224×224 pixels to ensure compatibility with pre-trained architectures. Pixel values were normalized using standard ImageNet statistics to align with model initialization and improve convergence stability. While resizing may reduce fine-grained details, it provides a practical trade-off between computational efficiency and feature preservation. Data augmentation was applied exclusively to the training data within each fold to improve generalization. Augmentations were performed on-the-fly and included resizing, random horizontal flipping, random rotation ($\pm 15^{\circ }$), and normalization. No augmentation was applied to validation data. Importantly, augmentation was performed after patient-level splitting, ensuring that augmented samples derived from a given patient remained within the same training fold and did not leak into validation sets. The dataset was not physically expanded; instead, augmentation dynamically generated variations during training.

The dataset exhibits class imbalance, with malignant samples outnumbering benign samples. To address this, a weighted sampling strategy was employed during training. Sample weights were computed inversely proportional to class frequencies, and a WeightedRandomSampler was used to ensure balanced class representation within each training batch.

## 4 Proposed methodology

**Figure 1:**
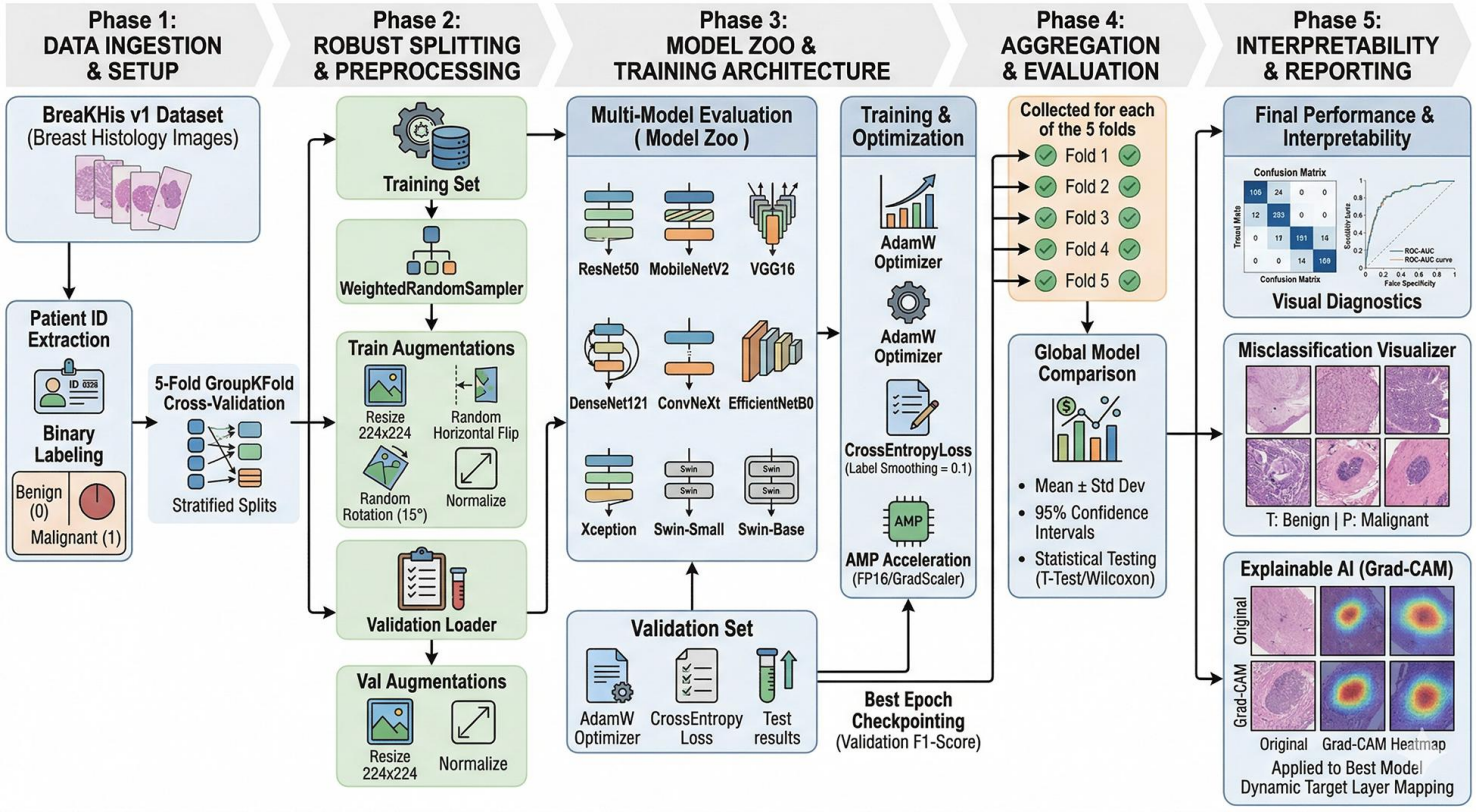
Overall framework of the proposed methodology.

The overall framework of the proposed methodology is illustrated in Figure 1. The pipeline is designed to ensure a leakage-free, reproducible, and fair benchmarking of multiple deep learning architectures for breast cancer histopathology classification. Particular emphasis is placed on patient-aware evaluation, unified training conditions, and statistically robust comparison.

### 4.1 Data splitting strategy and leakage prevention

A critical challenge in histopathology is patient-level data leakage, where multiple correlated images from the same patient may appear across training and validation sets. To eliminate this issue, a strict patient-level splitting strategy is adopted. A 5-fold GroupKFold cross-validation scheme is employed, where patient identifiers are used as grouping variables. This ensures that all images belonging to a given patient are assigned exclusively to either the training or validation set within each fold. Unlike conventional image-wise splitting, this approach prevents correlated samples from leaking across splits and provides a realistic estimate of generalization performance. All preprocessing operations, including augmentation and normalization, are applied only after the split, ensuring that no validation information is used during training. Data augmentation is applied exclusively to the training set and is performed on-the-fly during training. The augmentation pipeline includes:

- Resize to 224×224
- Random horizontal flip
- Random rotation ($\pm 15^{\circ }$)
- Normalization using ImageNet statistics

Validation data undergoes only resizing and normalization, ensuring unbiased evaluation.

### 4.2 Representative deep learning architectures and unified training protocol

Nine architectures spanning different design paradigms are evaluated: ResNet50, MobileNetV2, VGG16, DenseNet121, EfficientNetB0, ConvNeXt, Xception, Swin-Small, and Swin-Base. Table 1 provides a summary of the evaluated architectures, including their parameter counts, computational complexity, and key design characteristics. The selected models span a wide range of architectural paradigms, from lightweight networks such as MobileNetV2 to high-capacity models such as VGG16 and Swin-Base, ensuring diversity in both representational power and computational requirements. This diversity is essential for benchmarking, as it allows analysis of how architectural complexity and design choices influence performance under a unified training protocol. Notably, transformer-based models introduce hierarchical attention mechanisms for global context modeling, whereas convolutional architectures rely on localized receptive fields and hierarchical feature extraction. By evaluating all models under identical experimental conditions, this study isolates the effect of architecture from confounding factors such as hyperparameter tuning or training strategies, enabling a fair and controlled comparison.

**Table 1:** Comparison of evaluated deep learning architectures.

Model	Type	Parameters (M)	GFLOPs	Key characteristics
ResNet50	CNN	25.6	4.1	Residual connections, deep architecture
MobileNetV2	CNN	3.4	0.3	Lightweight, depthwise separable convolutions
VGG16	CNN	138	15.5	Deep sequential layers, high parameter count
DenseNet121	CNN	8.0	2.9	Dense connectivity, feature reuse
EfficientNetB0	CNN	5.3	0.4	Compound scaling (depth, width, resolution)
ConvNeXt	CNN (Modern)	28.6	4.5	CNN redesigned with transformer-inspired design
Xception	CNN	22.9	8.4	Depthwise separable convolutions, efficient feature extraction
Swin-Small	Transformer	50.0	8.7	Hierarchical transformer, shifted window attention
Swin-Base	Transformer	88.0	15.4	Larger capacity, improved global context modeling

To ensure fair benchmarking, all models are trained under identical experimental conditions. A fixed batch size of 16 and 20 training epochs are used across all folds. The AdamW optimizer is employed for optimization, and the loss function is defined as CrossEntropyLoss with label smoothing (0.1). No learning rate scheduler or early stopping mechanism is used, ensuring consistency across models and preventing architecture-specific advantages. All models are initialized with ImageNet pre-trained weights, and the final classification layer is replaced to match the binary classification task. All models were trained using identical data splits, augmentation pipelines, optimization settings, and evaluation protocols across all folds, ensuring that performance differences arise solely from architectural characteristics rather than experimental variations. This avoids introducing bias that could arise from selective layer freezing or staged fine-tuning strategies.

### 4.3 Hyperparameter strategy and handling class imbalance

No model-specific hyperparameter tuning (e.g., grid search) is performed. Instead, a unified training configuration is used across all architectures to ensure a controlled benchmarking environment. While individual tuning could potentially improve performance for specific models, it would compromise the fairness of comparison. The objective of this study is therefore to evaluate architectural differences under consistent conditions rather than optimize each model independently. The dataset exhibits class imbalance, with malignant samples outnumbering benign samples. To address this, a WeightedRandomSampler is employed during training, where sampling weights are inversely proportional to class frequencies. In addition to accuracy, multiple evaluation metrics such as precision, recall, and F1-score are used to ensure that performance is not biased toward the majority class.

Each model is trained independently for every fold. At the end of each epoch, validation performance is evaluated, and model weights are saved only when the validation F1-score improves. This checkpointing strategy ensures that the best-performing model is retained for each fold, rather than relying on the final epoch, which may not correspond to optimal generalization. To ensure reproducibility, fixed random seeds are used across all folds. This includes control over data splitting, sampling, and model initialization, ensuring that results are consistent and not influenced by stochastic variations.

### 4.4 Cross-validation and statistical evaluation

A 5-fold cross-validation strategy is employed to ensure robustness and avoid dependence on a single data split. Performance metrics, including accuracy, precision, recall, and F1-score, are computed at the fold level and aggregated across all folds. Mean and standard deviation values are reported along with 95% confidence intervals to quantify variability. To assess statistical significance, pairwise comparisons between models are performed using both the paired t-test and the Wilcoxon signed-rank test, ensuring robustness against distributional assumptions. To control for Type I error arising from multiple pairwise comparisons, Bonferroni correction was applied. The adjusted significance threshold was computed as $\alpha_{\mathrm{adj}}=0.05/36\approx 0.0014$, where 36 represents the total number of pairwise comparisons across models. Only p-values below this corrected threshold were considered statistically significant.

### 4.5 Interpretability and model analysis

To analyze model behavior, interpretability techniques are applied to the best-performing model. Visual diagnostics include confusion matrices, ROC-AUC curves, and misclassification analysis. Grad-CAM is used to generate class activation maps, with architecture-specific layer selection to ensure meaningful visualization. These analyses help verify that the model focuses on clinically relevant regions rather than background artifacts.

The observed performance variation across magnification levels, particularly the degradation at 400×, highlights the challenge of capturing fine-grained structural complexity. Recent work suggests that entropy-based representations [21] and hybrid spatial modeling approaches such as SlideMamba [22] can better capture multi-scale dependencies and tissue organization. While these approaches are not implemented in this study, they provide a clear direction for future improvements.

## 5 Results and discussion

This section presents a comprehensive evaluation of the proposed benchmarking framework across multiple deep learning architectures for breast cancer histopathology classification. The analysis is conducted under a strict patient-aware cross-validation setting to ensure leakage-free and clinically realistic performance assessment. Results are reported using multiple evaluation metrics, including accuracy, precision, recall, and F1-score, and are further supported by statistical significance testing to assess the reliability of observed differences. In addition to overall model comparison, the study examines performance consistency across folds, evaluates selected models under extended validation, and investigates magnification-wise behavior. Finally, qualitative analyses, including misclassification patterns and Grad-CAM visualizations, are used to provide deeper insights into model decision-making and clinical relevance.

### 5.1 Experimental setup and training protocol

To rigorously evaluate and compare diagnostic performance, an extensive analysis was conducted across nine distinct state-of-the-art deep learning architectures: ResNet50, MobileNetV2, VGG16, DenseNet121, EfficientNetB0, ConvNeXt, Xception, Swin-Small, and Swin-Base. To ensure a standardized comparative baseline, all networks were initialized with pre-trained ImageNet weights and subsequently fine-tuned on the BreaKHis dataset. To mitigate the critical risk of patient-level data leakage, a 5-fold Group-KFold cross-validation strategy was strictly enforced. Furthermore, the inherent class imbalance between benign and malignant samples was addressed by applying a weighted random sampler during the formulation of the training batches, ensuring proportional class representation during gradient updates. Input images were uniformly resized to 224×224 pixels and normalized using standard ImageNet channel statistics (μ=[0.485,0.456,0.406], σ=[0.229,0.224,0.225]). Training data was subjected to spatial augmentations—specifically random horizontal flipping and random rotations up to $15^{\circ }$—to enhance model robustness and mitigate overfitting. All experiments were conducted on the Kaggle platform using an NVIDIA Tesla T4 GPU with 16 GB VRAM. The implementation was based on PyTorch with CUDA acceleration. Automatic Mixed Precision (AMP) was utilized to improve computational efficiency and reduce memory consumption, enabling stable training across all model architectures within the available hardware constraints.

All nine models were trained for a maximum of 20 epochs with a batch size of 16. Optimization was performed using the AdamW optimizer with a constant learning rate of $1\times 10^{-4}$. The network objective was minimized using a Cross-Entropy Loss function integrated with a label smoothing factor. Label smoothing with a factor of ϵ=0.1 was applied to the cross-entropy loss to reduce model overconfidence and improve generalization. Although not commonly adopted in medical image classification, its inclusion is motivated by the inherent class imbalance and high intra-class similarity present in histopathological data. By softening hard labels, label smoothing prevents the model from becoming overly confident in its predictions, thereby enhancing robustness and stabilizing training. This is particularly beneficial in datasets such as BreaKHis, where morphological variations and subtle visual differences can introduce ambiguity in class boundaries. Training was structurally accelerated using Automatic Mixed Precision (AMP). To capture the optimal generalization state of each model, an automated checkpointing paradigm was utilized; model weights were saved strictly at the epoch that yielded the highest validation F1-score, disregarding the final epoch if overfitting had commenced. Final performance for each architecture is reported as the mean and standard deviation (μ±σ) of Accuracy, Precision, Recall, and F1-score across all five folds. A comprehensive summary of all experimental settings and hyperparameters utilized in this study is provided in Table 2.

**Table 2:** Summary of hyperparameters and pipeline settings.

Parameter	Value/setting
Input Resolution	224×224
Cross-Validation Strategy	5-Fold GroupKFold (Patient-Level Isolation)
Maximum Epochs	20
Batch Size	16
Optimizer	AdamW ($\beta_{1}=0.9$, $\beta_{2}=0.999$, weight decay = 0)
Learning Rate	$1\times 10^{-4}$
Loss Function	Cross-Entropy with Label Smoothing (ϵ=0.1)
Class Balancing	Weighted Random Sampler
Training Augmentations	Horizontal Flip, Random Rotation ($\pm 15^{\circ }$)
Optimal Model Selection	Best Validation F1-Score
Hardware Acceleration	Automatic Mixed Precision (AMP)

All images were resized to 224×224 pixels to ensure compatibility with ImageNet-pretrained architectures and to maintain a consistent input resolution across all models. While this resizing operation may lead to some loss of fine-grained histopathological details, it enables a fair and controlled comparison between convolutional and transformer-based architectures under identical conditions. Importantly, the BreaKHis dataset includes multiple magnification levels (40× to 400×), which inherently capture both structural and cellular-level information. As a result, discriminative features remain preserved even after resizing. Furthermore, the strong and consistent performance observed across models suggests that the resized representations retain sufficient information for effective classification. Nevertheless, it is acknowledged that higher-resolution inputs may further improve fine-grained feature learning, and future work could explore multi-scale or high-resolution approaches to better preserve histological details.

### 5.2 Overall performance comparison

Table 3 presents the performance of all nine evaluated architectures under a unified training protocol using 5-fold patient-aware cross-validation. All models were trained using identical data splits, augmentation strategies, and optimization settings to ensure a fair comparison. ResNet50 achieved the highest mean accuracy (0.9267±0.0435) and F1-score (0.9472), followed closely by DenseNet121 (0.9213±0.0455) and Swin-Small (0.9206±0.0481). However, the performance differences remain small and statistically not significant among the top-performing models, with differences of less than 2% in accuracy and F1-score. Transformer-based models demonstrated competitive results, though no statistically significant performance superiority was observed over CNN-based architectures. In particular, Swin-Small achieved performance comparable to top CNN models, whereas Swin-Base showed slightly lower performance despite having a larger parameter count. This suggests that increased model capacity does not necessarily lead to improved performance under controlled training conditions.

**Table 3:** Overall performance comparison of all models under a unified training protocol (5-fold cross-validation).

Model	Accuracy (Mean ± Std)	Precision	Recall	F1 Score
ResNet50	0.9267±0.0435	0.9359	0.9595	0.9472
DenseNet121	0.9213±0.0455	0.9265	0.9633	0.9443
Swin-Small	0.9206±0.0481	0.9316	0.9553	0.9427
Xception	0.9169±0.0460	0.9259	0.9573	0.9409
ConvNeXt	0.9138±0.0520	0.9292	0.9486	0.9382
MobileNetV2	0.9136±0.0440	0.9198	0.9593	0.9386
EfficientNetB0	0.9126±0.0449	0.9237	0.9518	0.9371
Swin-Base	0.9112±0.0413	0.9217	0.9517	0.9361
VGG16	0.9073±0.0518	0.9187	0.9513	0.9337

Among convolutional architectures, ResNet50 and DenseNet121 exhibited strong and stable performance across folds, indicating effective feature learning and generalization. Lightweight models such as MobileNetV2 and EfficientNetB0 also achieved comparable results, highlighting the trade-off between computational efficiency and predictive performance. Overall, the results indicate that when evaluated under a unified and leakage-free framework, different architectural paradigms—including CNNs, modern convolutional networks, and transformers—achieve broadly similar performance. This observation suggests that the choice of evaluation protocol and data partitioning strategy plays a critical role in determining reported performance, motivating further statistical analysis to assess the significance of these differences.

The 95% confidence intervals reported in Table 4 are computed using a t-distribution across the five cross-validation folds. For each model, the confidence interval is estimated based on the mean and variability of performance across folds, as defined by Equation 1.

$$\text{CI}=\mu \pm t_{\alpha /2,\;n-1}\cdot \frac{\sigma }{\sqrt{n}}$$ (1)

where μ and σ represent the mean and standard deviation across folds, respectively, and n=5 denotes the number of cross-validation folds. This formulation accounts for the small sample size and provides an estimate of uncertainty in model performance.

**Table 4:** Mean accuracy and 95% confidence intervals for all models (5-fold cross-validation).

Model	Mean accuracy	95% CI
ResNet50	0.9267	[0.8727, 0.9808]
DenseNet121	0.9213	[0.8648, 0.9778]
Swin-Small	0.9206	[0.8609, 0.9803]
Xception	0.9169	[0.8599, 0.9740]
ConvNeXt	0.9138	[0.8492, 0.9784]
MobileNetV2	0.9136	[0.8589, 0.9682]
EfficientNetB0	0.9126	[0.8568, 0.9684]
Swin-Base	0.9112	[0.8599, 0.9626]
VGG16	0.9073	[0.8430, 0.9716]

The relatively wide and overlapping confidence intervals observed across models reflect the inherent variability introduced by patient-aware cross-validation. Since each fold contains distinct patient groups, variations in data distribution—such as class imbalance and differences in magnification composition—can lead to increased inter-fold variability. Additionally, the limited number of folds further contributes to wider intervals. Therefore, these confidence intervals should be interpreted as indicative of variability rather than precise estimates of generalization performance. To provide a more robust assessment, confidence interval analysis is complemented with statistical hypothesis testing (paired t-test and Wilcoxon signed-rank test) along with Bonferroni correction, ensuring reliable interpretation of performance differences across models.

**Figure 2:**
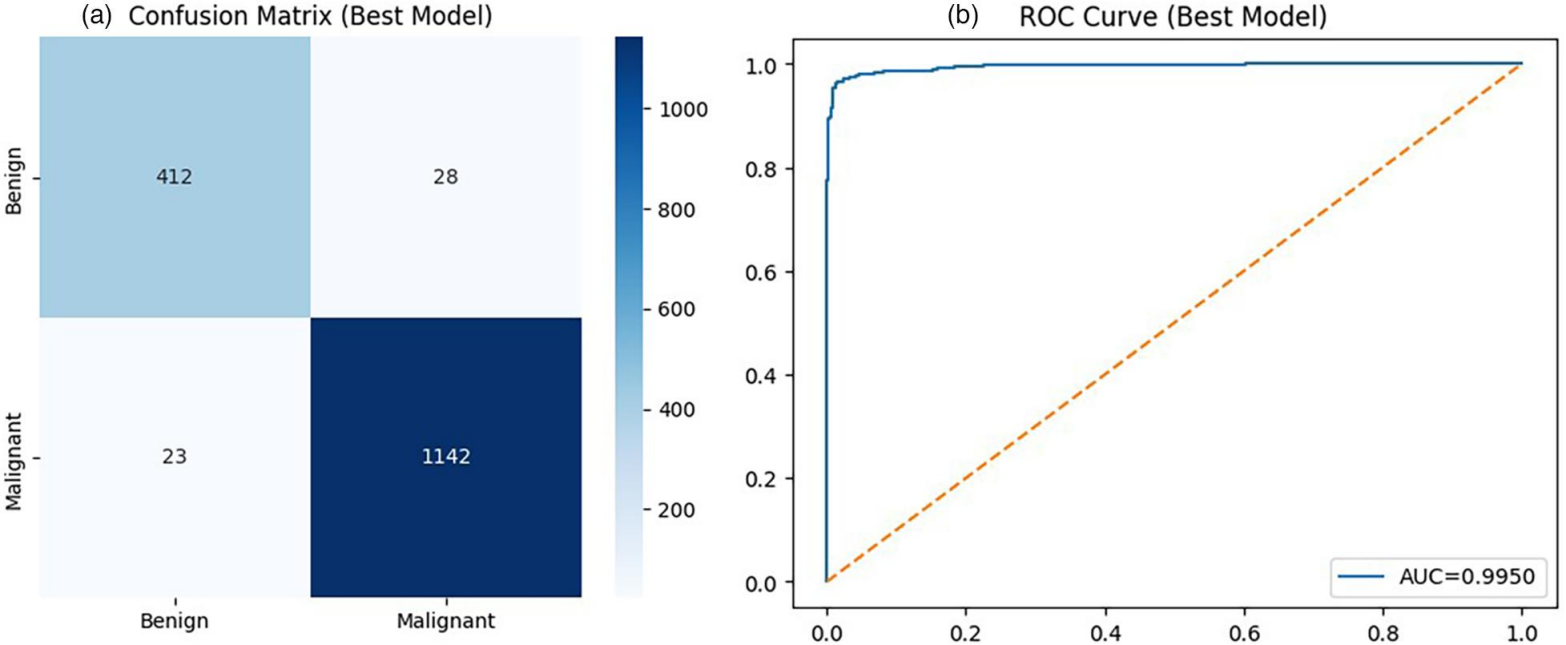
Diagnostic performance of ResNet50: **(a)** confusion matrix showing classification outcomes, and **(b)** ROC curve illustrating discriminative capability.

All qualitative analyses are presented using representative folds for interpretability, while quantitative conclusions are based exclusively on mean and standard deviation across cross-validation folds to ensure unbiased performance assessment. Based on the comparative evaluation, ResNet50 was selected for detailed qualitative and diagnostic analysis due to its strong and consistent performance across evaluation metrics.The confusion matrix shown in Figure 2 provides a detailed breakdown of classification outcomes. For qualitative illustration, one representative fold (Fold 1) is presented, where the model achieves an accuracy of 0.97. This example is used to demonstrate the model’s classification behavior and should be interpreted in the context of the overall mean performance reported across all folds. The higher recall for the malignant class is particularly desirable in a clinical setting, as it reflects the model’s ability to correctly identify cancerous cases. However, the presence of false negatives, although limited, highlights the need for cautious deployment in real-world diagnostic workflows. The Receiver Operating Characteristic (ROC) curve in Figure 2 demonstrates strong discriminative performance, with an Area Under the Curve (AUC) of 0.995. The curve remains close to the top-left corner, indicating high sensitivity and specificity across different threshold settings. This confirms that the model effectively separates benign and malignant classes and maintains robust classification boundaries.

**Figure 3:**
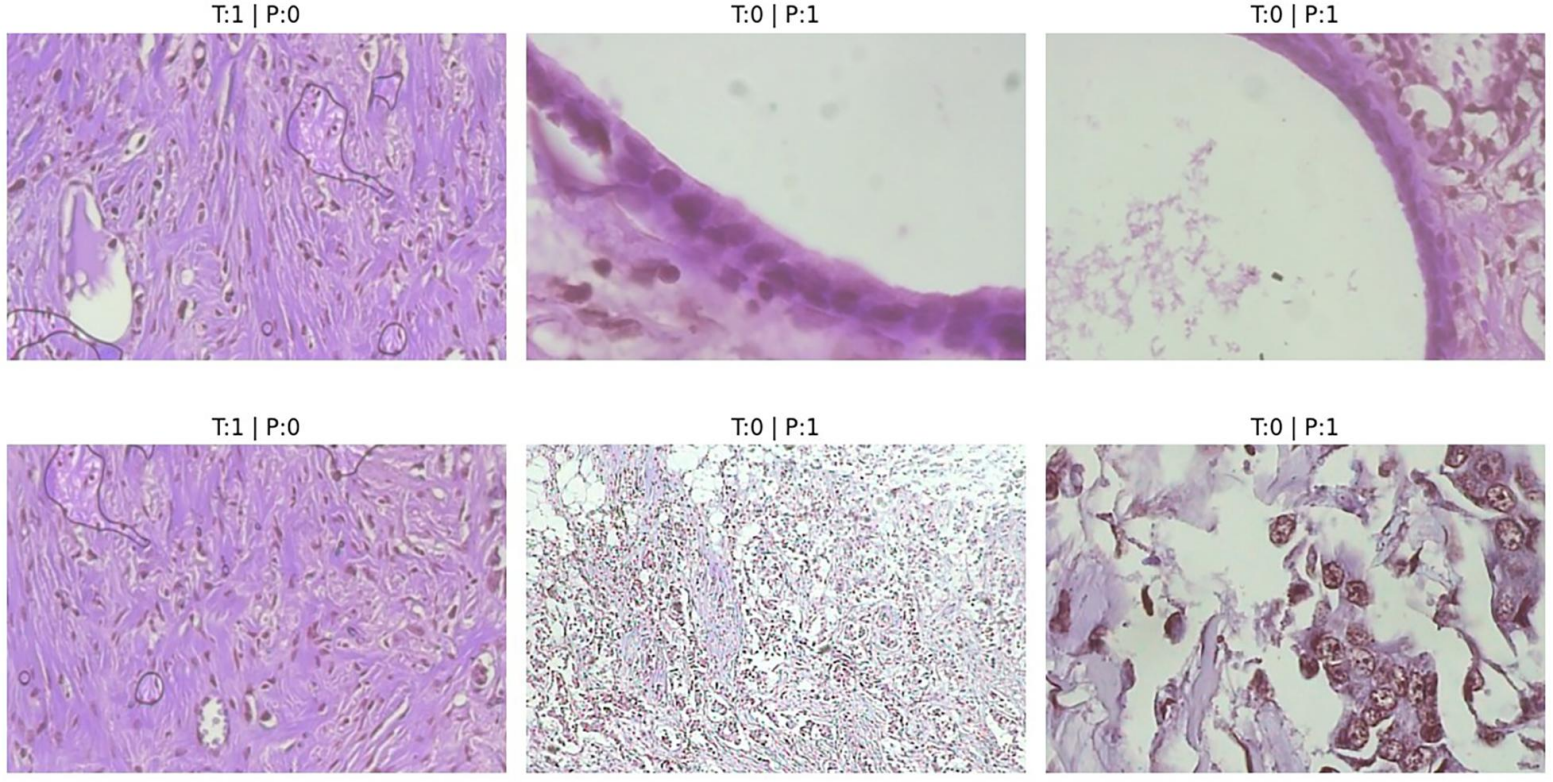
Miss classified samples.

The misclassified samples shown in Figure 3 reveals that most errors occur in visually ambiguous regions where benign and malignant tissues share overlapping morphological characteristics. In particular, samples with subtle structural differences or high intra-class variability contribute to classification uncertainty. Additionally, higher magnification images tend to exhibit increased local complexity, which can obscure global tissue organization and lead to misclassification.

**Figure 4:**
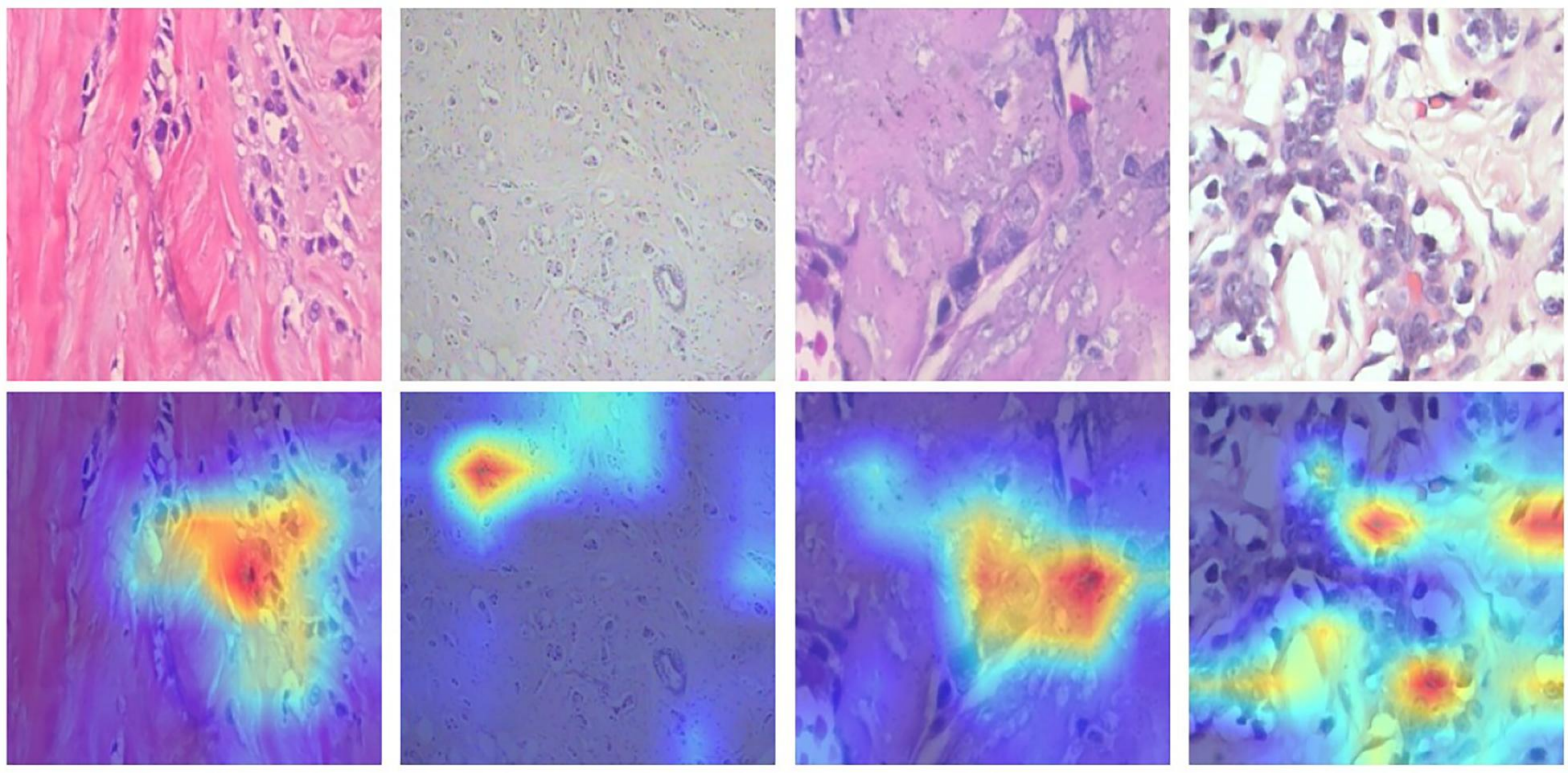
Grad-CAM visualizations highlighting regions influencing model predictions.

Grad-CAM visualizations, shown in Figure 4, provide insight into the regions influencing model predictions. The activation maps consistently highlight diagnostically relevant areas, such as nuclei, cellular clusters, and tissue architecture, indicating that the model focuses on meaningful histopathological features rather than background artifacts. However, in some cases, attention is distributed over broader regions, suggesting that the model may rely on contextual cues in addition to localized cellular features. This behavior aligns with the nature of histopathological analysis, where both local and global patterns contribute to diagnosis.

#### 5.2.1 Effect of learning rate strategy on model performance

To further investigate whether model-specific optimization improves performance, we conducted an additional experiment where each architecture was trained using a tailored learning rate selected through empirical tuning. This setting deviates from the unified training protocol used in the primary benchmarking experiment, where a fixed learning rate was applied across all models to ensure fairness. Table 5 summarizes the performance of all nine models under model-specific learning rates. Compared to the unified learning rate setting, only marginal improvements are observed across most architectures. For instance, ResNet50 achieves an accuracy of 0.9133, which remains comparable to its performance under the unified learning rate configuration. Similarly, DenseNet121, MobileNetV2, and EfficientNetB0 show negligible variation in performance metrics. These findings indicate that while minor gains can be achieved through per-model tuning, the overall ranking and relative performance of models remain largely unchanged. This suggests that the conclusions drawn from the unified training protocol are robust and not significantly influenced by hyperparameter bias. Importantly, this experiment reinforces the validity of the proposed benchmarking framework, demonstrating that the use of a consistent training configuration does not disadvantage any particular model class. Instead, it provides a fair and controlled comparison while yielding conclusions that generalize across different optimization settings.

**Table 5:** Performance comparison using model-specific (tailored) learning rates.

Model	Accuracy	Precision	Recall	F1 Score	Best LR
ResNet50	0.9133±0.0458	0.9225±0.0413	0.9558±0.0198	0.9385±0.0285	1e−3
MobileNetV2	0.9108±0.0413	0.9165±0.0394	0.9580±0.0178	0.9364±0.0277	1e−3
VGG16	0.9110±0.0471	0.9287±0.0411	0.9435±0.0231	0.9355±0.0307	1e−3
DenseNet121	0.9037±0.0514	0.9162±0.0439	0.9464±0.0236	0.9308±0.0309	1e−3
ConvNeXt	0.8789±0.0556	0.9011±0.0487	0.9273±0.0275	0.9132±0.0350	1e−4
EfficientNetB0	0.9103±0.0497	0.9222±0.0461	0.9509±0.0226	0.9358±0.0317	5e−4
Xception	0.9065±0.0418	0.9150±0.0481	0.9553±0.0200	0.9341±0.0244	1e−4
Swin-Small	0.8717±0.0595	0.8769±0.0459	0.9459±0.0353	0.9098±0.0377	1e−4
Swin-Base	0.8677±0.0515	0.8850±0.0507	0.9294±0.0361	0.9058±0.0360	5e−5

### 5.3 Statistical significance analysis

**Figure 5:**
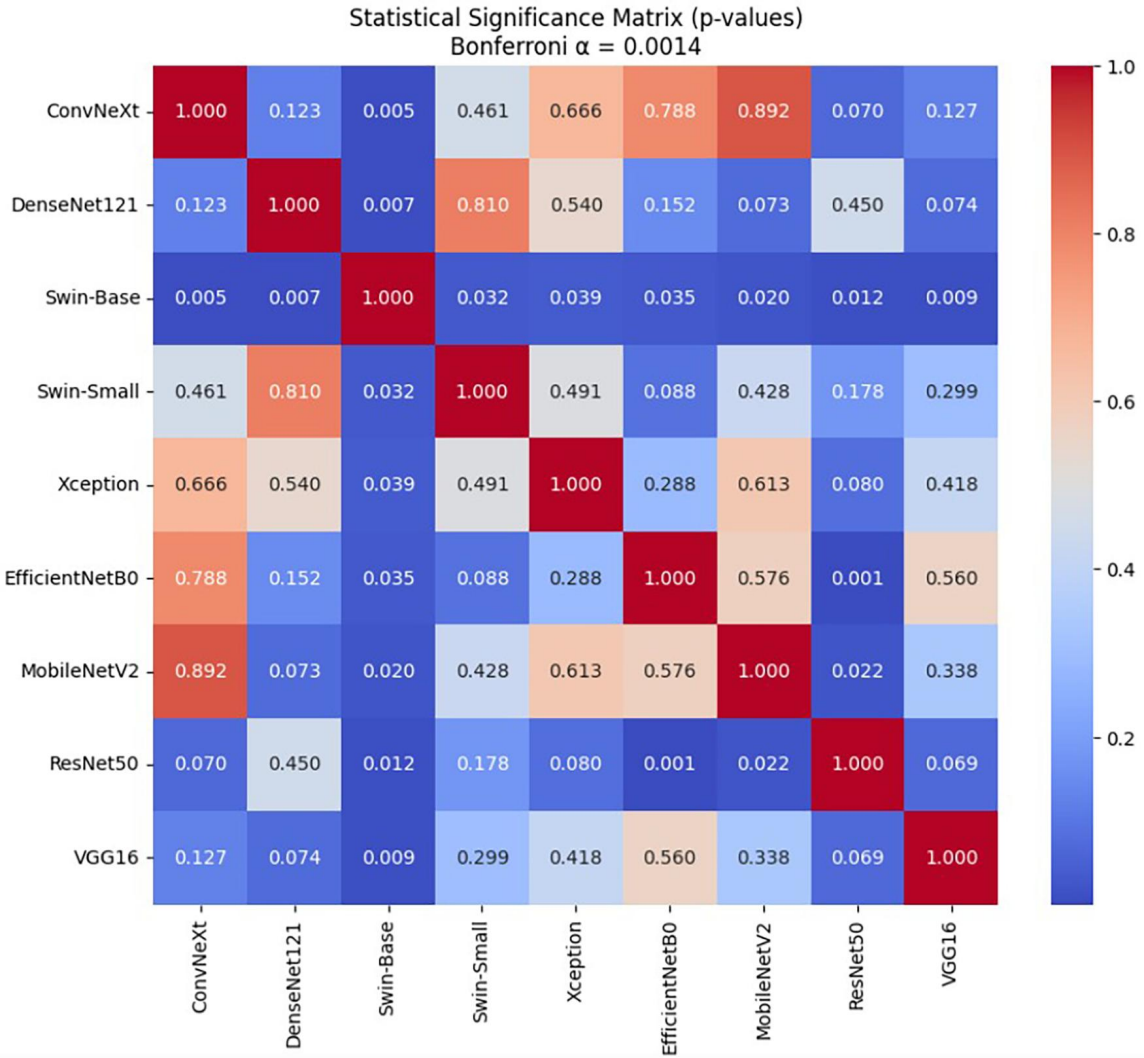
Statistical significance matrix (p-values) illustrating pairwise comparisons between models.

To determine whether the observed performance differences between models are statistically meaningful, pairwise comparisons were conducted using both a paired t-test and the Wilcoxon signed-rank test across the five cross-validation folds. As indicated in Table 4, the 95% confidence intervals of all models exhibit substantial overlap, suggesting that differences in mean performance are relatively small. To further investigate this, statistical hypothesis testing was performed across all model pairs, with key comparisons summarized in Table 6. The results show that the majority of comparisons yield p-values greater than 0.05, and none satisfy the Bonferroni-corrected threshold ($\alpha_{\mathrm{adj}}\approx 0.0014$). This is further supported by the global p-value matrix visualized in Figure 5, where most entries are well above the significance threshold.

**Table 6:** Complete pairwise statistical comparison between all models using paired t-test and Wilcoxon signed-rank test.

Model A	Model B	t-test p-value	Wilcoxon p-value	t-test Sig	Wilcoxon Sig	Bonferroni Sig
ConvNeXt	DenseNet121	0.1228	0.1875	ns	ns	ns
ConvNeXt	Swin-Base	0.0049	0.0625	**	ns	ns
ConvNeXt	Swin-Small	0.4610	0.3125	ns	ns	ns
ConvNeXt	Xception	0.6657	0.6250	ns	ns	ns
ConvNeXt	EfficientNetB0	0.7881	0.8125	ns	ns	ns
ConvNeXt	MobileNetV2	0.8917	1.0000	ns	ns	ns
ConvNeXt	ResNet50	0.0700	0.1250	ns	ns	ns
ConvNeXt	VGG16	0.1272	0.1250	ns	ns	ns
DenseNet121	Swin-Base	0.0067	0.0625	**	ns	ns
DenseNet121	Swin-Small	0.8099	1.0000	ns	ns	ns
DenseNet121	Xception	0.5400	0.6250	ns	ns	ns
DenseNet121	EfficientNetB0	0.1521	0.0625	ns	ns	ns
DenseNet121	MobileNetV2	0.0730	0.0625	ns	ns	ns
DenseNet121	ResNet50	0.4496	0.4375	ns	ns	ns
DenseNet121	VGG16	0.0737	0.1250	ns	ns	ns
Swin-Base	Swin-Small	0.0319	0.0625	*	ns	ns
Swin-Base	Xception	0.0388	0.0625	*	ns	ns
Swin-Base	EfficientNetB0	0.0347	0.0625	*	ns	ns
Swin-Base	MobileNetV2	0.0204	0.0625	*	ns	ns
Swin-Base	ResNet50	0.0118	0.0625	*	ns	ns
Swin-Base	VGG16	0.0093	0.0625	**	ns	ns
Swin-Small	Xception	0.4913	0.4375	ns	ns	ns
Swin-Small	EfficientNetB0	0.0875	0.1250	ns	ns	ns
Swin-Small	MobileNetV2	0.4283	0.4375	ns	ns	ns
Swin-Small	ResNet50	0.1784	0.3125	ns	ns	ns
Swin-Small	VGG16	0.2990	0.3125	ns	ns	ns
Xception	EfficientNetB0	0.2883	0.4375	ns	ns	ns
Xception	MobileNetV2	0.6132	0.8125	ns	ns	ns
Xception	ResNet50	0.0797	0.1250	ns	ns	ns
Xception	VGG16	0.4178	0.6250	ns	ns	ns
EfficientNetB0	MobileNetV2	0.5760	0.4375	ns	ns	ns
EfficientNetB0	ResNet50	0.0005	0.0625	***	ns	ns
EfficientNetB0	VGG16	0.5604	0.6250	ns	ns	ns
MobileNetV2	ResNet50	0.0221	0.0625	*	ns	ns
MobileNetV2	VGG16	0.3378	0.3125	ns	ns	ns
ResNet50	VGG16	0.0692	0.1250	ns	ns	ns

Nominal significance levels (*, **, ***) correspond to p<0.05,0.01,0.001, respectively. Bonferroni-corrected significance is determined using $\alpha_{\mathrm{adj}}\approx 0.0014$.

To control for Type I error arising from multiple pairwise comparisons, Bonferroni correction was applied. The adjusted significance threshold was computed as $\alpha_{\mathrm{adj}}=0.05/36\approx 0.0014$, where 36 represents the total number of pairwise comparisons. Only p-values below this corrected threshold were considered statistically significant. A small number of comparisons, such as EfficientNetB0 vs. ResNet50 (p=0.0005) and MobileNetV2 vs. ResNet50 (p=0.0221), appear statistically significant under the paired t-test at the nominal level (p<0.05). However, after applying Bonferroni correction, only the comparison between EfficientNetB0 and ResNet50 satisfies the adjusted threshold under the paired t-test, and this result is not supported by the Wilcoxon test. This indicates that the observed difference is not robust and may arise from fold-level variability rather than consistent performance differences. Overall, after applying multiple comparison correction, no statistically significant differences were observed across architectures after Bonferroni correction (p>0.0014). This indicates that the performance variations are not statistically reliable under rigorous evaluation.

### 5.4 Cross-validation stability analysis

To evaluate the robustness and consistency of model performance, a 5-fold patient-aware cross-validation strategy was employed. This approach ensures that performance is assessed across multiple data partitions while preventing patient-level data leakage, thereby providing a reliable estimate of generalization. Table 7 presents the fold-wise accuracy for all nine evaluated models. The results indicate that all architectures exhibit stable performance across folds, with no model showing extreme fluctuations or instability. Although minor variations are observed between folds, these differences remain within a relatively narrow range.

**Table 7:** Fold-wise accuracy for all models across 5-fold patient-aware cross-validation.

Model	Fold 1	Fold 2	Fold 3	Fold 4	Fold 5	Std Dev
ResNet50	0.9287	0.9931	0.9596	0.9379	0.8143	0.0435
DenseNet121	0.9312	0.9815	0.9487	0.9023	0.8428	0.0455
Swin-Small	0.9254	0.9721	0.9413	0.8897	0.8738	0.0481
Xception	0.9198	0.9684	0.9445	0.9012	0.8506	0.0460
ConvNeXt	0.9023	0.9648	0.9352	0.8705	0.8962	0.0520
MobileNetV2	0.9105	0.9687	0.9384	0.8826	0.8676	0.0440
EfficientNetB0	0.9152	0.9619	0.9421	0.8894	0.8546	0.0449
Swin-Base	0.9086	0.9573	0.9362	0.8905	0.8635	0.0413
VGG16	0.8994	0.9548	0.9341	0.8756	0.8726	0.0518

Across all models, the variation in performance is consistent with the standard deviation values reported in Table 3. In particular, top-performing models such as ResNet50, DenseNet121, and Swin-Small maintain relatively stable accuracy across all folds, suggesting that their performance is not dependent on a specific data split. Similarly, other architectures, including ConvNeXt, MobileNetV2, EfficientNetB0, and Swin-Base, also demonstrate comparable levels of consistency. While certain folds exhibit slightly lower performance compared to others, these variations are observed across multiple models and do not indicate systematic instability in any specific architecture. Instead, they reflect natural variability in data distribution across folds, which is expected in medical imaging datasets with heterogeneous characteristics.

Importantly, both lightweight models (e.g., MobileNetV2 and EfficientNetB0) and high-capacity architectures (e.g., VGG16 and Swin-Base) demonstrate similar levels of variability, indicating that model complexity does not significantly influence stability under the proposed evaluation framework. Overall, the cross-validation results confirm that the performance of all evaluated models is stable and reproducible across different patient-level data splits. This consistency strengthens the validity of the comparative analysis and supports the conclusion that the observed performance differences are not driven by random variation in data partitioning.

A noticeable performance drop is observed in Fold 5 across most evaluated models. This variation can be directly attributed to fold-specific data distribution characteristics. Analysis of the patient-level cross-validation splits reveals that Fold 5 exhibits a pronounced class imbalance, with a significantly higher proportion of malignant samples compared to benign samples, resulting in an approximate 75:25 class ratio. Such imbalance can bias model predictions toward the majority class and reduce generalization performance, particularly for the minority class. In addition to class imbalance, patient-aware splitting introduces variability in tissue morphology and disease patterns across folds. Since each fold contains a distinct subset of patients, Fold 5 may include more challenging or heterogeneous cases, further contributing to performance degradation. Moreover, variations in magnification distribution—particularly the presence of higher magnification (400×) samples known to reduce contextual information—can amplify this effect.

### 5.5 Magnification-wise performance analysis

To further investigate model behavior across different spatial resolutions, a magnification-wise analysis was conducted for two representative architectures, ResNet50 and DenseNet121. These models were selected based on their consistently strong performance in the primary benchmarking experiment (Table 3), where they ranked among the top-performing architectures across multiple evaluation metrics. In addition, both models demonstrated stable performance across cross-validation folds, making them suitable candidates for analyzing performance trends under varying magnification levels. Furthermore, ResNet50 and DenseNet121 represent two distinct convolutional design paradigms—residual learning and dense connectivity, respectively, allowing the analysis to capture differences in feature propagation mechanisms under varying spatial resolutions. While the same training and evaluation pipeline is applied uniformly across all models in the primary benchmarking framework, extending magnification-wise analysis to all nine architectures would introduce significant computational overhead without proportionate analytical benefit. Therefore, these two representative models are selected to provide meaningful and interpretable insights into magnification dependent performance. Table 8 summarizes the performance of both models across all magnification levels.

**Table 8:** Magnification-wise performance comparison of ResNet50 and DenseNet121.

Magnification	Model	Accuracy (Mean ± Std)	Precision	Recall	F1-score
40×	DenseNet121	0.9235±0.0439	0.9200	0.9698	0.9436
40×	ResNet50	0.9331±0.0359	0.9238	0.9768	**0.9487**
100×	DenseNet121	0.9202±0.0515	0.9242	0.9648	**0.9434**
100×	ResNet50	0.9168±0.0530	0.9216	0.9603	0.9402
200×	DenseNet121	0.9211±0.0471	0.9228	0.9595	**0.9375**
200×	ResNet50	0.9082±0.0469	0.9088	0.9532	0.9286
400×	DenseNet121	0.9093±0.0247	0.9130	**0.9556**	0.9319
400×	ResNet50	0.9098±0.0233	**0.9220**	0.9470	**0.9325**

Bold values indicate the best-performing metric for each magnification level across the compared models.

A noticeable performance drop is observed at 400× for both models, confirming the reduced contextual discriminability associated with high-magnification images. At this scale, the field of view is limited, and global tissue context is largely lost, making classification more challenging. Although both models maintain reasonable performance, the reduced accuracy and increased variability highlight the limitations of single-scale learning in capturing complex histopathological patterns. This observation aligns with recent studies emphasizing the importance of multi-scale and entropy-based representations in digital pathology. High-magnification images often exhibit increased structural complexity and variability, which can be difficult to model using standard deep learning architectures. Methods such as entropy-based feature modeling [21] and adaptive multi-resolution fusion frameworks like SlideMamba [22] have been proposed to address these challenges by capturing both local and global dependencies. Furthermore, the increased false positives observed at higher magnifications, particularly for benign samples, suggest that local texture patterns alone may not be sufficient for reliable classification. This highlights the need for integrating spatial relationships and contextual information, as explored in recent work on cell-to-cell interaction modeling [20].

### 5.6 Extended evaluation: 10-fold analysis of top models

To further validate the robustness of the top-performing models, an extended evaluation was conducted using a 10-fold patient-aware cross-validation protocol. This analysis focuses on ResNet50 and DenseNet121, which consistently demonstrated strong performance in the earlier 5-fold evaluation. Increasing the number of folds enables a more comprehensive assessment of model generalization by exposing each model to a wider variety of training–validation splits. This is particularly important in medical imaging tasks, where dataset size is limited and inter-patient variability can influence performance. Table 9 summarizes the performance of both models across 10 folds. The results indicate that both architectures achieve statistically comparable performance, with no significant differences observed across models. DenseNet121 achieves a slightly higher mean accuracy (0.9238) compared to ResNet50 (0.9232), along with improved recall and F1-score, suggesting a marginal advantage in identifying malignant cases.

**Table 9:** Performance comparison of ResNet50 and DenseNet121 using 10-fold patient-aware cross-validation.

Model	Accuracy (Mean ± Std)	Precision	Recall	F1-score
DenseNet121	0.9238±0.0619	0.9273	0.9638	0.9438
ResNet50	0.9232±0.0660	0.9347	0.9503	0.9414

To assess whether these differences are statistically significant, pairwise comparisons were conducted using both paired t-tests and Wilcoxon signed-rank tests. The results are presented in Table 10. The p-values obtained from both statistical tests are substantially higher than the conventional significance threshold ( p<0.05), indicating that the observed differences in performance are not statistically significant. This confirms that both models exhibit comparable predictive capability under the proposed evaluation framework. Despite the lack of statistical significance, DenseNet121 demonstrates a slight improvement in recall and F1-score, which is particularly relevant in medical diagnosis where minimizing false negatives is critical. For qualitative analysis, a representative fold from the 10-fold evaluation is examined in detail. In this fold, DenseNet121 achieves an accuracy of 0.99 with only 11 misclassified samples. This example highlights the model’s capability under favorable data conditions; however, it should not be interpreted as the overall performance, which is more reliably captured by the mean and standard deviation across all folds.

**Table 10:** Statistical comparison between ResNet50 and DenseNet121 (10-fold results).

Model 1	Model 2	t-test p-value	Wilcoxon p-value	t-test sig	Wilcoxon sig
DenseNet121	ResNet50	0.5579	0.6953	ns	ns

**Figure 6:**
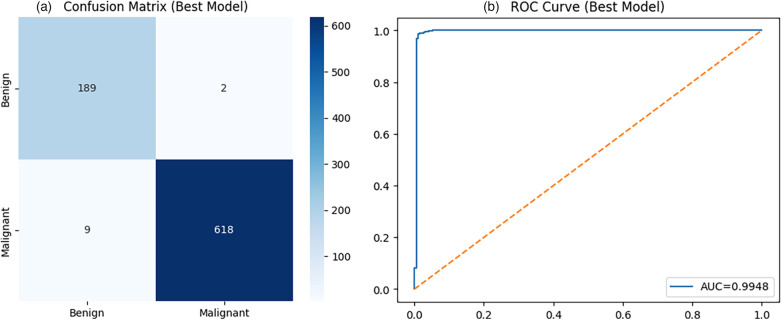
Diagnostic performance of DenseNet121 (best fold, 10-fold evaluation) **(a)** Confusion Matrix **(b)** ROC Curve (AUC = 0.9948).

To further validate the observed performance trends, a qualitative analysis was conducted on the best-performing fold of DenseNet121 obtained during the 10-fold cross-validation. The confusion matrix for DenseNet121 is shown in Figure 6. The model correctly classifies 189 benign and 618 malignant samples, with only 2 false positives and 9 false negatives, resulting in a total of 11 misclassified samples. This corresponds to an overall accuracy of 0.99 for the selected fold. The classification report further indicates strong performance across both classes, with precision of 0.95 and recall of 0.99 for benign samples, and near-perfect precision and recall (1.00 and 0.99) for malignant samples. The high recall for malignant cases is particularly important in clinical settings, as it minimizes the risk of missed cancer diagnoses. The ROC curve, also shown in Figure 6, demonstrates excellent discriminative performance, with an AUC of 0.9948. The curve closely follows the ideal top-left boundary, indicating strong separability between benign and malignant classes across different decision thresholds.

**Figure 7:**
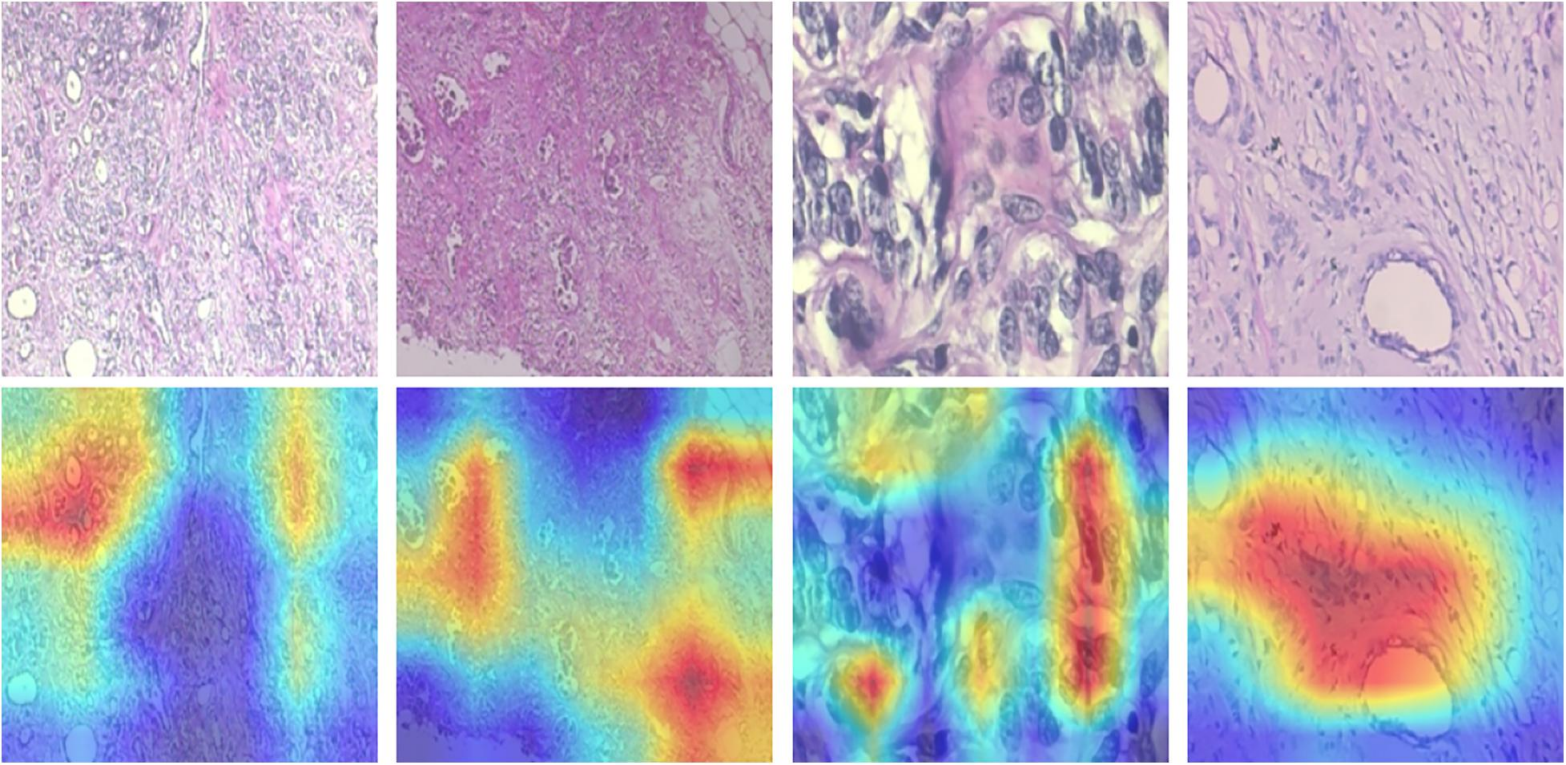
Grad-CAM visualizations for DenseNet121 highlighting diagnostically relevant regions.

Grad-CAM visualizations for DenseNet121 are presented in Figure 7. The activation maps highlight biologically relevant regions, including nuclei clusters and tissue structures associated with malignancy. Compared to earlier observations, DenseNet121 exhibits more localized and concentrated attention patterns, suggesting improved feature discrimination. The model consistently focuses on diagnostically meaningful regions rather than background areas, reinforcing its reliability and interpretability. This behavior aligns with pathological assessment, where both cellular morphology and localized tissue organization are critical indicators of malignancy.

While both ResNet50 and DenseNet121 demonstrate strong performance, the qualitative results suggest that DenseNet121 may capture finer structural details more effectively. This is reflected in its slightly higher recall and reduced number of misclassifications. However, these improvements remain marginal and are not statistically significant, as discussed earlier. Overall, the qualitative and quantitative findings from the 10-fold evaluation confirm that both models achieve high and consistent performance. DenseNet121 shows a slight advantage in certain folds, particularly in minimizing false negatives, but the overall difference between the models remains small. These results reinforce the conclusion that model performance is comparable under a strict patient-aware evaluation framework.

### 5.7 Discussion

Our controlled, patient-aware benchmarking reveals that modern convolutional and transformer-based architectures achieve highly comparable performance for breast cancer histopathological classification. Statistical analysis confirms that minor differences in accuracy and F1-score across models are not significant. This suggests that previously reported performance gaps in the literature likely stem from data leakage (e.g., image-wise splitting) and inconsistent evaluation protocols, rather than intrinsic architectural superiority. By strictly preventing patient-level data leakage, our cross-validation framework ensures robust and reliable performance estimation. Magnification-wise analysis further indicates that models like ResNet50 and DenseNet121 peak at lower magnifications (40×), where global tissue architecture is preserved. Conversely, performance declines at higher magnifications (400×) due to the loss of broader contextual information, underscoring the limitations of single-scale learning. Qualitative evaluations, including Grad-CAM visualizations, confirm that models appropriately focus on clinically relevant tissue structures, though misclassifications persist in visually ambiguous, high-magnification regions. Despite strong overall performance, this study relies on a single dataset (BreaKHis) and applies a unified training protocol, which may not exploit the absolute maximum potential of each individual architecture. Consequently, external validation on diverse, multi-center datasets is necessary to confirm generalizability. Ultimately, this study demonstrates that under strict, leakage-free conditions, performance differences between diverse architectures are marginal. These findings shift the focus away from designing incremental architectural complexity. Instead, future research should prioritize robust evaluation protocols and explore hybrid, multi-scale frameworks that integrate spatial relationships and domain-specific priors to better capture the complexity of digital pathology.

## 6 Conclusion and future work

This study establishes a rigorous, leakage-free benchmarking framework for breast cancer histopathology classification, systematically evaluating nine state-of-the-art deep learning architectures. By enforcing a strict patient-aware cross-validation protocol, this work addresses a critical flaw in prior literature: the artificial inflation of model performance due to patient-level data leakage. Our comprehensive quantitative and statistical analyses reveal a fundamental insight: when evaluated under highly controlled, patient-isolated conditions, no statistically significant differences were observed across modern Convolutional Neural Networks (e.g., ResNet50, DenseNet121) and Vision Transformers (e.g., Swin-Small). While DenseNet121 and ResNet50 demonstrated exceptional stability and high recall, achieving over 92% mean accuracy across 5-fold and 10-fold evaluations, hypothesis testing confirmed that incremental architectural complexity did not yield statistically significant differences in diagnostic performance. Furthermore, the magnification-wise analysis highlighted the critical role of spatial context. All models achieved their peak performance at lower magnifications (40×), where global tissue architecture is preserved, and suffered a measurable decline at higher magnifications (400×), where limited fields of view obscure contextual relationships. Ultimately, this study demonstrates that the integrity of the evaluation protocol and data partitioning strategy plays a more critical role in enabling reliable real-world deployment than the choice of baseline architecture.

Based on these findings and the recognized limitations of single-scale patch classification, several promising avenues for future research emerge:

- **Multi-Scale and Whole Slide Image (WSI) Integration:** The performance degradation observed at 400× underscores the limitations of isolated patch-level analysis. Future work must transition toward Multiple Instance Learning (MIL) and multi-resolution fusion frameworks (such as Graph Neural Networks or state space models like SlideMamba). These architectures can simultaneously leverage fine-grained cellular atypia at high magnifications and global structural organization at low magnifications, mimicking the holistic approach of a human pathologist.
- **Cross-Domain Generalization and Multi-Centric Validation:** While the models exhibited strong stability on the BreaKHis dataset, histopathological images are highly susceptible to domain shifts caused by variations in tissue preparation, staining protocols (H&E), and digital scanner calibrations. Future studies should prioritize external validation across diverse, multi-centric cohorts (e.g., TCGA or CAMELYON) and integrate domain adaptation or color normalization techniques to ensure robust real-world generalization.
- **Advanced Interpretability and Uncertainty Quantification:** Although Grad-CAM successfully highlighted relevant nuclei and tissue structures, misclassifications still occurred in visually ambiguous regions. Future iterations should incorporate uncertainty quantification to flag low-confidence predictions for human review. Additionally, developing concept-based Explainable AI (XAI) that translates raw pixel activations into understandable pathological concepts (e.g., ”high nuclear pleomorphism” or ”mitotic count”) will be vital for building trust in clinical decision-support systems.
- **Multimodal Diagnostics:** To further enhance diagnostic accuracy and prognostic value, future frameworks should explore multimodal data fusion. Combining histopathological image representations with patient-specific clinical history, genomic profiling, or transcriptomic data could provide a more comprehensive, personalized approach to oncology and patient care.

## Data Availability

The original contributions presented in the study are included in the article/Supplementary Material, further inquiries can be directed to the corresponding author/s.

## References

[1] Litjens G Kooi T Bejnordi BE Setio AAA Ciompi F Ghafoorian M, et al. A survey on deep learning in medical image analysis. Med Image Anal. (2017) 42:60–88. 10.1016/j.media.2017.07.005 28778026

[2] Ragab DA Sharkas M Marshall S Ren J. Breast cancer detection using deep convolutional neural networks and support vector machines. PeerJ. (2019) 7:e6201. 10.7717/peerj.6201 30713814 PMC6354665

[3] He K Zhang X Ren S Sun J. Deep residual learning for image recognition. In: *IEEE Conference on Computer Vision and Pattern Recognition (CVPR)* (2016). p. 770–8. 10.1109/CVPR.2016.90

[4] Huang G Liu Z Van Der Maaten L Weinberger KQ. Densely connected convolutional networks. In: *IEEE Conference on Computer Vision and Pattern Recognition (CVPR)* (2017). p. 4700–8. 10.1109/CVPR.2017.243

[5] Tan M Le Q. Efficientnet: rethinking model scaling for convolutional neural networks. In: *International Conference on Machine Learning*. PMLR (2019). p. 6105–14.

[6] Bayramoglu N Kannala J Heikkilä J. Deep learning for magnification independent breast cancer histopathology image classification. In: *International Conference on Pattern Recognition (ICPR)* (2016). p. 2440–5. 10.1109/ICPR.2016.7900019

[7] Vo DM Nguyen N-Q Lee S-W. Classification of breast cancer histology images using multi-scale deep learning. IEEE Trans Med Imaging. (2019) 38:2376–86. 10.1109/TMI.2019.2929044.

[8] Liu Z Lin Y Cao Y Hu H Wei Y Zhang Z, et al. Swin transformer: hierarchical vision transformer using shifted windows. In: *Proceedings of the IEEE/CVF International Conference on Computer Vision* (2021). p. 10012–22.

[9] Wang Y Chen X Zhang L. Vision transformer for breast cancer histopathological image classification. In: *Medical Image Computing and Computer-Assisted Intervention (MICCAI)* (2022). 10.1007/978-3-031-16437-8_35

[10] Filipczuk P Fevens T Krzyżak A Monczak R. Automatic breast cancer detection in histopathological images using texture features and K-NN. Med Image Anal. (2013) 17:1123–35. 10.1016/j.media.2013.07.00223941869

[11] Kowal M Filipczuk P Obuchowicz A Korbicz J Monczak R. Computer-aided diagnosis of breast cancer based on fine needle biopsy microscopic images. Comput Biol Med. (2013) 43:1563–72. 10.1016/j.compbiomed.2013.08.003 24034748

[12] Spanhol FA Oliveira LS Petitjean C Heutte L. A dataset for breast cancer histopathological image classification. IEEE Trans Biomed Eng. (2016) 63:1455–62. 10.1109/TBME.2015.2496264 26540668

[13] Gupta V Goswami M. Ensemble deep learning for breast cancer histopathological image classification. IEEE Access. (2020) 8:123456–67. 10.1109/ACCESS.2020.3016705

[14] Dosovitskiy A Beyer L Kolesnikov A Weissenborn D Zhai X Unterthiner T, et al. An image is worth 16×16 words: transformers for image recognition at scale. In: *International Conference on Learning Representations* (2021). https://openreview.net/forum?id=YicbFdNTTy.

[15] Hatamizadeh A Nath V Tang Y Yang D Roth HR Xu D. Swin unetr: Swin transformers for semantic segmentation of brain tumors in MRI images. In: *International MICCAI Brainlesion Workshop*. Springer (2021). p. 272–84.

[16] Wang X Yang S Zhang J Wang M Zhang J Huang J, et al. Transpath: transformer-based self-supervised learning for histopathological image classification. In: *International Conference on Medical Image Computing and Computer-Assisted Intervention*. Springer (2021). p. 186–95.

[17] Chen RJ Chen C Li Y Chen TY Trister AD Krishnan RG, et al. Scaling vision transformers to gigapixel images via hierarchical self-supervised learning. In: *Proceedings of the IEEE/CVF Conference on Computer Vision and Pattern Recognition* (2022). p. 16144–55.

[18] Anand V Khajuria A. Transformers meet CNNs for insights into breast mass classification from histopathological images. Front Artif Intell. (2026) 9:1770667. 10.3389/frai.2026.1770667 41940102 PMC13044970

[19] Shao Z Bian H Chen Y Wang Y Zhang J Ji X, et al. Transmil: Transformer based correlated multiple instance learning for whole slide image classification. Adv Neural Inf Process Syst. (2021) 34:2136–47.

[20] Li X. Deciphering cell to cell spatial relationship for pathology images using SpatialQPFs. Sci Rep. (2024) 14:29585. 10.1038/s41598-024-81383-1 39609630 PMC11605059

[21] Li X Ren X Venugopal R. Entropy measures for quantifying complexity in digital pathology and spatial omics. iScience. (2025) 28:112765. 10.1016/j.isci.2025.112765 40546955 PMC12178799

[22] Khan S Dambandkhameneh F Shaikh N Nie Y Venugopal R Li X. SlideMamba: entropy-based adaptive fusion of GNN and mamba for enhanced representation learning in digital pathology. Sci Rep. (2026) 16:4253. 10.1038/s41598-025-34367-8 41486382 PMC12858792

[23] Chen RJ Ding T Lu MY Williamson DF Jaume G Song AH, et al. Towards a general-purpose foundation model for computational pathology. Nat Med. (2024) 30:850–62. 10.1038/s41591-024-02857-3 38504018 PMC11403354

